# The austral-most record of the genus *Haemagogus* Williston (Diptera: Culicidae)

**DOI:** 10.1590/0037-8682-0222-2019

**Published:** 2019-12-20

**Authors:** Gisella Obholz, Fernando Diez, Germán San Blas, Gustavo Rossi

**Affiliations:** 1 Universidad Nacional de La Pampa, Facultad de Ciencias Exactas y Naturales, Santa Rosa, La Pampa, Argentina.; 2 Consejo Nacional de Investigaciones Científicas y Técnicas (CONICET), Buenos Aires, Argentina.; 3 Centro de Estudios Parasitológicos y de Vectores, CCT La Plata, CONICET UNLP, La Plata, Buenos Aires, Argentina.

**Keywords:** New record, Haemagogus spegazzinii, Yellow fever, La Pampa, Argentina

## Abstract

**INTRODUCTION:**

The genus *Haemagogus* Williston is restricted to Central America and North and middle of South America and it includes numerous species of yellow fever virus vectors.

**METHODS:**

Adult female and larvae mosquitoes were collected using hand aspirators and dipper and pipette, respectively.

**RESULTS:**

The first record of a species of *Haemagogus* and particularly of *Haemagogus spegazzinii* was from La Pampa, Argentina. With this registry, the number of species found in La Pampa province rises to 18.

**CONCLUSIONS:**

New information on breeding sites for the species and implications of this new record suggest a possible extension of distribution in the near future.

The genus *Haemagogus* Williston, 1896 includes numerous species of yellow fever virus vectors. Their immature stages are found mainly in tree holes, but they can also be found in artificial containers; the adults generally inhabit savannas, forests, and cultivated areas^1^. To conduct control programs, it is necessary to know the distribution of mosquito species, mainly those with public health implications, which is essential to determine areas of potential risk of transmission of diseases^2^. *Haemagogus* is restricted to North and middle of South America, Central America, and the Caribbean islands (from Jamaica to Martinique) with a single species recorded in the Nearctic, in Texas, USA. In South America, its distribution includes north to central regions of the subcontinent, from Venezuela to the north of Argentina, except for the Pacific coast of the Gulf of Guayaquil (Ecuador) and certain elevations of the Andes^3^ ^,^ ^4^ ^,^ ^5^. There are some mosquito genera, such as *Toxorhynchites*, *Orthopodomyia*, *Haemagogus*, *Limatus*, *Isostomyia*, *Onirion*, *Sabethes*, *Trichoprosopon, Wyeomyia,* and certain subgenera of *Anopheles, Aedes,* and *Culex,* whose species exclusively use phytotelmata as breeding sites*.* Among the phytotelmata most used by mosquitoes are bamboo, tree holes, floral bracts such as woody phytotelmata, and herbaceous phytotelmata such as Apiaceae, Araceae, and Bromeliaceae^6^ while several of the species also do so in artificial containers. Among the species of *Haemagogus*, there is single record of phytotelmata on *Prosopis*, *Prosopis juliflora* as the breeding site of *Haemagogus equinus* ^7^. In Argentina, 4 species are cited out of the 28 species described for the genus *Haemagogus*: *Hg*. (*Con*.) *leococelaenus* Dyar and Shannon, *Hg*. (*Hag*.) *capricornii* Lutz, *Hg*. (*Hag*.) *janthinomys* Dyar and *Hg*. (*Hag*.) *spegazzinii* Brèthes^6^. These species are specialists, that is, they use phytotelmata as their only breeding site, although only two species have been reported breeding in tree holes: *Hg. leucocelaenus* in Misiones and *Hg. spegazzinii* in Salta, Córdoba^6^, and Chaco^8^; in all cases, the host plants were not identified.

*Hg. spegazzinii* extends in South America from eastern and southeastern Brazil to Paraguay, northern Argentina, and eastern Bolivia and Ecuador, Venezuela^3^, and Colombia^9^ ^*^. In Argentina, its distribution covers the provinces of Catamarca, Chaco, Córdoba, Corrientes, Formosa, Jujuy, Salta, San Luis, Santa Fe, Santiago del Estero, Tucumán, Mendoza, and Misiones^8^ ^,^ ^10^.

La Pampa province is located in the central region of Argentina. This province has an arid temperate weather with 300-850 mm of annual precipitation and annual temperatures ranging between 14 and 16 °C^11^. One of the biogeographical provinces represented in the region is the Pampean province situated in the north-eastern region^12^. Only 17 species of mosquitoes are cited, and none of them belong to *Haemagogus* ^13^. In the present study, new distributional records are provided, and *Prosopis caldenia* is recorded as a new host plant to *Haemagogus spegazzinii* breeding sites. 

One adult female mosquito was collected using hand-held aspirators on humans and was kept in a labeled plastic pot with cotton, paper, and naphthalene. Three mosquito larvae were collected by dipper and pipette, from a tree hole of *Prosopis caldenia* Burkart (Figure 1). Mosquitoes in the immature stages were kept in a labeled plastic bottle and brought to the laboratory for identification and reared up to one male and two females (Figure 2). Material obtained was identified based on morphological keys^3^. The abbreviations of genus and subgenus follow the approach of Reinert^14^.

**FIGURE 1:**
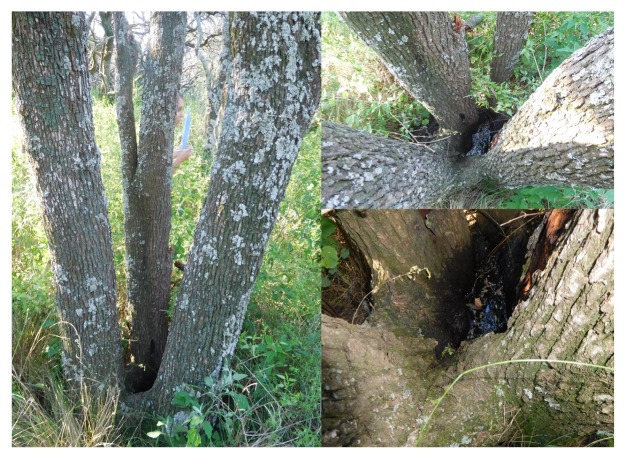
Breeding site of *Haemagogus spegazzinii* in *Prosopis caldenia*.

**FIGURE 2:**
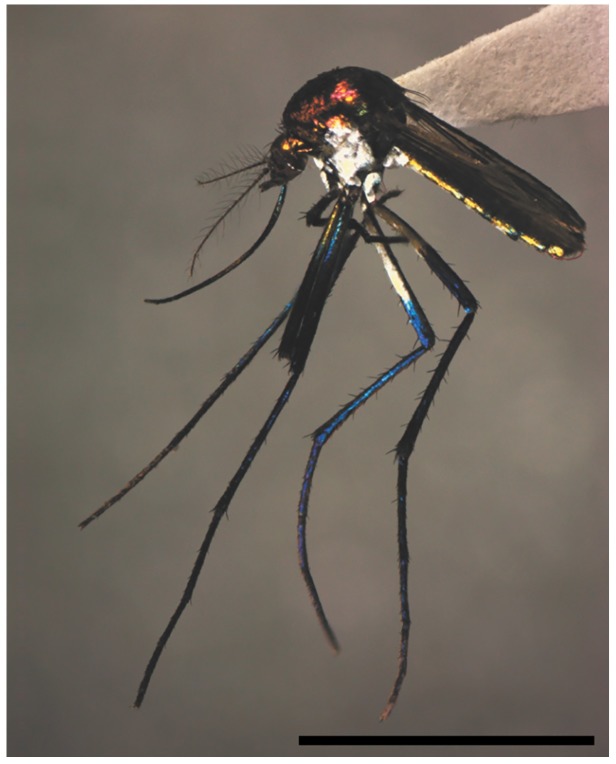
Photo of female *Haemagogus spegazzinii*. Scale bar: 10 mm.

Material Examined: La Pampa, Eduardo Castex, 35° 50′ 24.82′′ S, 64° 31′ 50.23′′ W (Figure 3), Feb. 2016. One male, two females MLPDipC 4733 a and b, and 1 larva.

**FIGURE 3:**
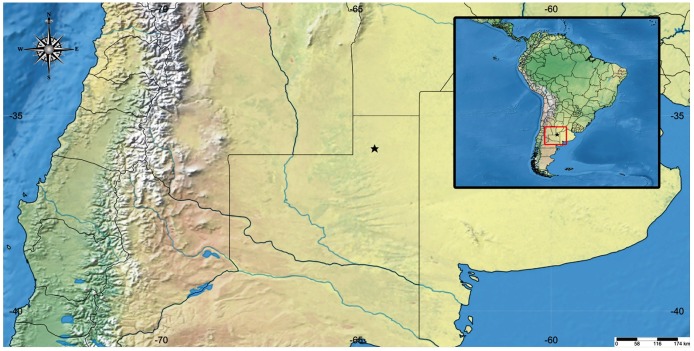
Map of the georeferenced point where *Haemagogus spegazzinii* was found.

Liria & Navarro^4^ performed a study on the potential distribution of the species of *Haemagogus* and concluded that La Pampa could be a province of distribution of *Hg. spegazzinii* but with low occurrence probability. Despite this species being collected in La Pampa, climate conditions in that year were special with heavy rains registered between January and February 2016 (120 and 141.5 mm, respectively). Furthermore, collections during following years were nil. We think this could mean the population is not established in the region, but because we found larvae, it means they were able to develop in this location. Despite the species not being established in the province, a change in climate conditions could make it suitable for its establishment in the future. This implication is of great medical importance for the province and Argentina; it could mean a probable future extension of distribution of this yellow fever vector in the country. 

Finally, in the present study, new distributional records are provided and *Prosopis caldenia* is recorded as a new host plant to *Haemagogus spegazzinii* breeding sites. This represents the first record of a species of *Haemagogus* and particularly of *Hg. spegazzinii* given that La Pampa, Argentina corresponds to the southernmost locality for species of the genus. With this registry, the number of species found in La Pampa province rises to 18. 

## Footnotes

* Comments: www.wbur.org mentioned this species from French Guiana and Trinidad and Tobago but neither country is mentioned in the literature and we did not find any specimens in the CEPAVE collection.
